# Serum procalcitonin elevation in critically ill patients at the onset of bacteremia caused by either gram negative or gram positive bacteria

**DOI:** 10.1186/1471-2334-8-38

**Published:** 2008-03-26

**Authors:** Pierre Emmanuel Charles, Sylvain Ladoire, Serge Aho, Jean-Pierre Quenot, Jean-Marc Doise, Sébastien Prin, Niels-Olivier Olsson, Bernard Blettery

**Affiliations:** 1 Service de Réanimation Médicale, Hôpital Le Bocage, C.H.U. de DIJON, France; 2 Service d'Epidémiologie et d'Hygiène Hospitalière, Hôpital Le Bocage, C.H.U. de DIJON, France; 3 Laboratoire d'Immunologie, Hôpital Le Bocage, C.H.U. de DIJON, France

## Abstract

**Background:**

In the ICU, bacteremia is a life-threatening infection whose prognosis is highly dependent on early recognition and treatment with appropriate antibiotics. Procalcitonin levels have been shown to distinguish between bacteremia and noninfectious inflammatory states accurately and quickly in critically ill patients. However, we still do not know to what extent the magnitude of PCT elevation at the onset of bacteremia varies according to the Gram stain result.

**Methods:**

Review of the medical records of every patient treated between May, 2004 and December, 2006 who had bacteremia caused by either Gram positive (GP) or Gram negative (GN) bacteria, and whose PCT dosage at the onset of infection was available.

**Results:**

97 episodes of either GN bacteremia (*n *= 52) or GP bacteremia (*n *= 45) were included. Procalcitonin levels were found to be markedly higher in patients with GN bacteremia than in those with GP bacteremia, whereas the SOFA score value in the two groups was similar. Moreover, in the study population, a high PCT value was found to be independently associated with GN bacteremia. A PCT level of 16.0 ng/mL yielded an 83.0% positive predictive value and a 74.0% negative predictive value for GN-related bacteremia in the study cohort (AUROCC = 0.79; 95% CI, 0.71–0.88).

**Conclusion:**

In a critically ill patient with clinical sepsis, GN bacteremia could be associated with higher PCT values than those found in GP bacteremia, regardless of the severity of the disease.

## Background

Bacteremia has long been associated with severity of illness, especially in an ICU setting [1,2]. Attributable mortality rates of around 35% have regularly been reported, but differences do exist and these depend on the pathogen [3,4]. Since inappropriate empirical antibiotherapy has proven harmful in the treatment of bloodstream infections (BSI), the bacteria responsible for the systemic infection needs to be identified [5]. In theory, current automated continuous-monitoring systems make it possible to detect bacterial growth only a few hours after the blood sample has been taken [6,7]. However, in everyday clinical practice, 12 to 24 hours are usually required to obtain the Gram stain result, once bacteria have been recovered from blood cultures. As a result, the outcome may be worse and the length of stay longer [8]. Other new approaches such as universal polymerase chain reaction (PCR) make it possible to identify bacteria quickly and reliably, but these are not routinely available in most centers [9]. Although knowledge of the clinical manifestations is invaluable, surrogate markers could help to identify the main human bacterial pathogens within the first hours of management of patients with bacteremia.

Serum procalcitonin (PCT) is a 116-amino-acid peptide, and elevated levels of this peptide are strongly associated with systemic bacterial infections [10]. Serum PCT measurement relies on a quick and routine lab test that has been reported to accurately differentiate between systemic bacterial infection and non-infectious acute inflammatory states, whereas white blood cells count (WBC) and serum C-reactive protein (CRP) failed to do so [11]. Moreover, it has been shown that the magnitude of PCT elevation closely correlates with outcome in critically ill patients [12].

Studies have previously reported a diference in PCT elevation depending on the involved pathogens, especially in cases of bacteremia, bacterial pneumonia and infective endocarditis [13-15]. However, up to now, only a few conflicting results regarding a PCT value that is able to distinguish between GP (Gram Positive) or GN (Gram Negative) bacterial infections have been provided, when considering critically ill patients with sepsis [12,16,17]. However, it has been clearly established that differences exist in the signaling pathways involved in the host's inflammatory response induced by the two bacterial species [18]. Since PCT elevation is thought to be closely related to the host's cytokine response to microbial challenge, we assumed that differences in magnitude according to the type of pathogen are present at the onset of bacteremia. PCT dosage is routinely performed in all patients with systemic inflammatory response syndrome admitted to our 15-bed ICU. Therefore, a retrospective cohort study was conducted.

## Methods

### Review of Medical Records

Each medical record was reviewed by a member of the medical staff (SL), unaware of the purpose of the study, following a standardized report sheet.

### Definitions

One episode of bacteremia was defined as the recovery of any bacterial species, in one or more blood cultures. Patients with polymicrobial cultures were not eligible. Neither were those with *Staphylococcus *non-*aureus *isolated in their blood cultures, unless the same species harboring the same antibiotic resistance pattern grew from at least 2 consecutive samples. Blood samples, obtained by venous puncture, were processed using the BACTEC system based on both standard aerobic and anaerobic media coupled with the 9240 automate (Beckton Dickinson Diagnostic Instrument System, Paramus, NJ, USA). Bacteria were identified using standard methods. The onset of bacteremia was defined as the day when the first positive blood culture was obtained. Two distinct episodes of bloodstream infection (BSI) were recorded for a patient if at least 6 days had elapsed between the 2 positive blood cultures, provided appropriate therapy had been implemented and significant clinical improvement had been obtained between the two episodes. This time interval was chosen since previously published data indicate that blood cultures become negative after an average of 2 days in patients with bacteremia receiving appropriate antimicrobial treatment.

### Study population

We reviewed the medical files of all consecutive patients admitted to our 11-bed medical ICU with subsequent bacteremia between 1^st ^May, 2004 and 31^st ^December, 2006. Only those with clinical sepsis (as defined by the American College of Chest Physicians/Society of Critical Care Medicine Consensus Conference) at the onset of BSI were included for further analysis, provided that at least one PCT dosage had been obtained within a 12-hour window surrounding the time when the first positive blood culture was drawn. The BSI episodes were then divided into 2 groups according to the pathogen isolated, which was either a Gram positive (GP group) or Gram negative (GN group) bacteria. As a result, if one patient presented 2 distinct BSI episodes, he was included in the same group twice or once in each group, according to the species isolated.

The following information was extracted from the medical file of each patient: (i) main clinical and epidemiological data on ICU admission, including age, gender, type of admission (admission was considered surgical in patients who had undergone surgery within the 30 days preceding the onset of BSI, and medical otherwise), and severity of illness on admission as defined by the Simplified Acute Physiology Score II (SAPS II); (ii) patient's characteristics at the onset of BSI, including WBC (cells/mm^3^), platelet count (cells/mm^3^), CRP value (mg/L), creatininemia (μmol/L), prothrombin time, septic condition (i.e., sepsis, severe sepsis or septic shock), and organ dysfunction expressed by the Sepsis-related Organ Failure Assessment (SOFA) score; (iii) infection source if known; (iv) antibiotics received within the 24 hours preceding bacteremia. In addition, all-cause overall ICU mortality was recorded.

### Measurements of PCT level

Plasma PCT assessment is usually performed in our ICU in patients with clinically suspected sepsis. The Kryptor^® ^immunoassay is used according to the manufacturer's instructions (Brahms, Hennigsdorf, Germany). The sensitivity of the assay is 0.06 ng/mL. Patients whose PCT measurement was unavailable or not performed within the 12 hours following the blood sampling were excluded from further analysis to avoid the risk of false-negative results.

### Statistical analysis

Values are expressed as mean ± SD unless otherwise stated. Continuous variables were compared thanks to the Mann Whitney U test. Categorical variables were compared using the Chi2-test. We then examined the independent contribution of factors that had been predictive of the Gram staining result in univariate analysis. In addition, conformity with the linear gradient of each continuous variable was checked. If the linear model was not appropriate to describe its variations, the variable was transformed according to the parcimonious rule (e.g., log_10_PCT was considered instead of PCT). The candidate variables were then manually entered into a logistical regression model if the associated regression coefficient had a *p *value less than 0.10 in univariate analysis, and then removed if a *p *value more than 0.05 was obtained in multivariate analysis. The number of these variables was limited to 6 with respect to the Harrell rule. The SOFA score was entered into the model regardless of the univariate analysis results, given its strong correlation with the PCT level [19,20].

The accuracy of serum PCT measurements at the onset of BSI for the distinction between GN and GP bacteremia was then expressed as the area under the corresponding receiver operating characteristic curve (AUROCC).

A *p *value < 0.05 was considered as statistically significant for all analyses. Stata software was used for all analyses (Stata Statistical Package, College Station, Tex., USA).

## Results

### Study population characteristics

Over the 32-month study period, 125 bacteremia episodes in 121 patients admitted to our ICU were recorded. Among these episodes, 1 was excluded because of the absence of clinical sepsis, 2 were excluded because of polymicrobial infections, 25 because of the lack of available PCT measurement at the onset of BSI (not done in time, *n *= 24; done in time but delayed analysis of the sample, *n *= 1). As a result, 97 bacteremia episodes in 92 patients were kept for final analysis.

Overall, the bacteremia episodes were encountered in 62 (63.9%) males and 35 (36.1%) females. The mean age was 64.8 (15.3) years. The mean SAPS II value at admission was 48.8 (18.6) points. The admission diagnosis was mainly medical (87 episodes out of 97 [89.6%]).

The excluded patients were not statistically different from the others regarding gender, age and admission diagnosis (data not shown). However, the SAPS II was significantly higher in excluded patients than in the study population (61.7 [20.8]; *p *= 0.002).

### Description of bloodstream infections

Among the 97 bacteremia episodes, 52 were caused by GN in 51 patients and 45 by GP bacterial species in 41 patients. Two patients successively presented one episode of GP bacteremia and one episode of GN bacteremia. Two patients presented 2 distinct episodes of GP bacteremia and 1 patient presented 2 distinct episodes of GN bacteremia. As defined previously, the first episode had resolved before the second occurred in all cases.

The proportions of medical admissions were similar in both groups (Table 1). However, there were more males in the GN group than in the GP group (71.1% and 55.6%, respectively; *p *= 0.115). In addition, patients with GP bacteremia were significantly younger than those with GN bacteremia (61.0 [14.1] and 68.2 [15.5] years old, respectively; *p *= 0.047). However, no difference was found in terms of SAPS II value on admission to the ICU (48.2 [18.4] and 48.8 [19.3], respectively).

**Table 1:** Baseline characteristics of the patients with bacteremia caused by either gram negative (GN) or gram positive (GP) bacteria.

Mean (SD) or number (%)	GN bacteremia *n *= 52	GP bacteremia *n *= 45	*p*
Age (year-old)	68.2 (15.5)	61.0 (14.1)	0.047
Male/female	71.1/28.9	55.6/44.4	0.115
Medical/surgical admission	88.5/11.5	88.9/11.1	0.947
SAPS II	48.8 (19.3)	48.2 (18.4)	0.870

SAPS: Simplified Acute Physiologic Score; GN: gram negative; GP: gram positive.

The most frequent bacterial species encountered were *Escherichia coli *(*n *= 26), *Staphylococcus aureus *(*n *= 19), *Streptococcus *species (*n *= 14), including *S. pneumoniae *(*n *= 5), and *Pseudomonas aeruginosa *(*n *= 10). The median time between the onset of clinical sepsis was 0.5 days (range: 0–7). The aforementioned bacteremia episodes that were excluded comprised 14 (50%) GP and 14 (50%) GN.

GP bacteremia tended to occur later than GN bacteremia during the stay in the ICU (9.0 [19.0] and 4.9 [7.2] days, respectively; *p *= 0.076) (Table 2). However, there was no difference between GP and GN bacteremia in terms of the frequency of septic shock at the onset of BSI. In addition, the source of infection was mainly the lung (34.6%), the abdomen (28.8%) or the urinary tract (25.0%) in the GN group whereas it was more likely to be the soft tissues (22.2%) in patients with GP bacteremia. Conversely, there was no difference when considering the proportion of inappropriate antibiotic treatments given within the 24 hours preceding bacteremia onset (2 out of 45 vs. 3 out of 52 patients with either GP or GN bacteremia, respectively).

**Table 2:** Description of the blood stream infections at the time of the first positive blood culture according to the gram stain result.

Mean (SD) or number (%)	GN bacteremia *n *= 52	GP bacteremia *n *= 45	*p*
Time elapsed from ICU admission (N. of days)	4.9 (7.2)	8.9 (18.8)	0.076
SOFA score	6.3 (4.5)	5.8 (3.0)	0.466
Platelet count (cell/mm3)	175,057 (20,839)	242,844 (20,880)	0.061
Creatininemia (μmol/L)	207.6 (176.0)	162.8 (148.4)	0.182
Prothrombin time (%)	57.9 (22.7)	64.7 (21.4)	0.144
Septic shock	24 (47.1)	22 (48.9)	0.789
Secondary sepsis Nosocomial sepsis	9 (17.3) 22 (42.3)	12 (26.7) 19 (42.2)	0.270 0.993
Infection source			0.001
Lung	18 (34.6)	10 (22.2)	0.184
Abdominal	15 (28.8)	5 (11.1)	0.053
Soft tissues	1 (2.0)	10 (22.2)	0.014
Urinary tract	13 (25.0)	3 (6.7)	0.022
Miscellaneous or unknown	5 (9.6)	17 (37.8)	0.480

ICU: Intensive Care Unit; SOFA: Sepsis-related Organ Failure Assessment; BSI: Blood Stream Infection; GN: Gram Negative; GP: Gram Positive.

### Measurement of serum PCT, CRP and WBC

**Figure 1:**
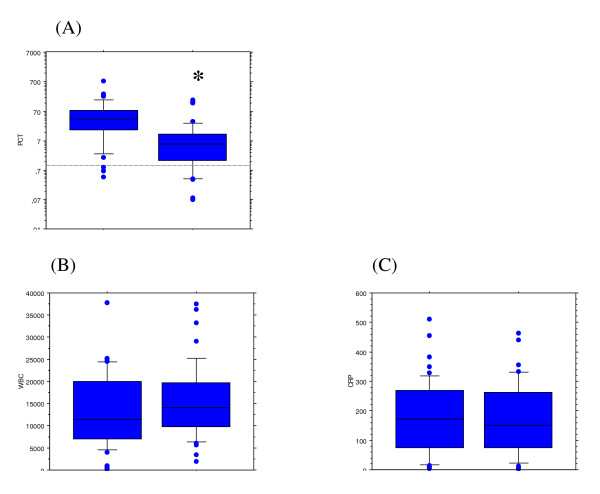
Serum procalcitonin (PCT) level (*Fig*. A), white blood cell count (WBC) (*Fig*. B) and C-reactive protein (CRP) level (*Fig*. C), at the onset of bacteremia caused by either gram negative (*left boxes*, GN; *n *= 52) or gram positive (*right boxes*, GP; *n *= 45) species in critically ill patients with clinical sepsis. Data are presented as box plots with median lines, 25- and 75-percentile boxes, and 10- and 90-percentile error bars. The circles represent the outliers. A log scale is used for the Y-axis in the Fig. A. * indicate *p *< 0.05 between GP and GN bacteremia.

Serum PCT levels at the time of bacteremia onset were markedly greater in the GN group than in the GP group (71.27 [116.42], median = 39.00 [range: 0.41–746.0] and 16.85 [37.34], median = 5.42 [range: 0.07–169.00] ng/mL, respectively; *p *= 0.003) (Figure 1). In contrast, the CRP values as well as the WBC in the two groups were similar. Very low levels of PCT (i.e., below the 0.5 ng/mL threshold) were found in 6 patients. Interestingly, bacteremia was caused by a GP bacteria in 5 of them. In addition, the source of infection was "superficial" (i.e., soft tissues or catheter) or unknown in all but one cases.

Given the results obtained in univariate analysis, the logistical regression model included the following variables: log_10_PCT level, time elapsed from ICU admission, platelet count, and some of the relevant infection sources were included as dichotomous variables (i.e., soft tissues [yes/no], abdominal [yes/no] and urinary tract [yes/no]) (Table 2). Given its clinical relevance, the SOFA score at the onset of bacteremia was also entered into the model [19,20]. Although episodes rather than patients were considered, age and gender, despite being demographic variables, were also entered into the multivariate analysis since obvious differences existed between GP and GN groups. In addition, only 5 out of 97 patients had repeated measures (e.g., presented 2 distinct BSI episodes).

Our model showed that PCT elevation was independently associated with the risk of GN bacteremia regardless of the infection source and the severity of the disease (Odds ratio = 4.17, 95% confidence interval, 2.08–8.33, *p *< 0.001) (Table 3).

**Table 3:** Multivariate analysis of the predictive factors for gram negative bacteremia in critically ill patients with clinical sepsis and positive blood cultures.

	Odds ratio	Variable type	95% CI		*p*
Soft tissue source of infection (yes)	9.10	dichotomous	1.09	100.0	0.042
Log_10_PCT	4.17	continuous	2.08-	8.33	<0.001

PCT: Procalcitonin; CI: Confidence Interval.

**Figure 2:**
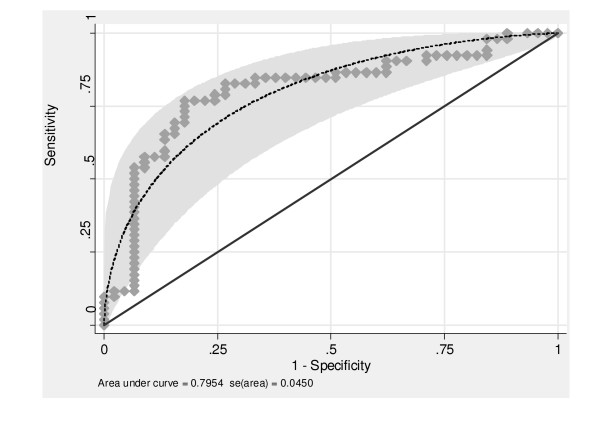
Receiver operating characteristic curve of serum procalcitonin (PCT) for the diagnosis of gram negative bacteremia in critically ill patients with clinical sepsis and blood cultures proven positive for bacterial species. Plain circles indicate PCT values and dashes represents the corresponding ROC curve assuming a normal distribution of the variable.

In addition, the corresponding ROC curve was constructed in order to assess to what extent PCT could differentiate between GP and GN bacteremia (Figure 2). The area under the ROC curve was 0.79 (95% CI, 0.71–0.88). A sensitivity of 75.0% and a specificity of 82.2%, a positive predictive value of 83.0% and a negative predictive value of 74.0% were achieved with a PCT cutoff value of 16.0 ng/mL (Table 4). The likelihood ratio of a positive test (LR+) was 4.21. The LR- was 0.30.

**Table 4:** Diagnostic accuracy of serum procalcitonin for the discrimination between gram positive and gram negative bacteremia in critically ill patients with clinical sepsis and positive blood cultures.

PCT cutoff value (ng/mL)	Sensitivity	Specificity	Positive predictive value	Negative predictive value	Likelihood ratio +	Likelihood ratio -
PCT > 16.0 95% CI	75.0% [61.0–86.0]	82.2% [68.0–92.0]	83.0% [69.0–92.0]	74.0% [60.0–85.0]	4.21 [1.91–10.7]	0.30 [0.15–0.57]

PCT: Procalcitonin; CI: Confidence Interval.

### Outcome

All-cause overall mortality in the ICU was 40.4% in the GN bacteremia group and 20.9% in the GP group (*p *= 0.070). PCT concentration at the onset of bacteremia was not found to be associated with a poor outcome (46.3 [61.5] in non-survivors *vs*. 47.0 [105.3] in survivors; *p *= 0.972).

## Discussion

Improving survival among critically ill patients with bacteremia relies on a number of interventions, among which the prompt administration of the appropriate antibiotics is obviously a key feature [5]. Thus, it has recently been shown that the so called "door-to-needle" time is a critical factor in the survival of patients with sepsis [21]. Clinical data as well as current guidelines generally help in the choice of empiric antibiotic treatment. However, some authors have reported that the antimicrobial regimen was changed in more than 25% of patients with BSI, and the Gram stain result was subsequently communicated to the physician in charge [22]. Surrogate markers are therefore needed to make the appropriate choice more rapidly.

Our findings suggest that the magnitude of PCT elevation could be significantly higher in patients with GN bacteremia than in those with GP bacteremia. Moreover no potential confounding variable was found. It is noteworthy that in previous published studies including critically ill patients with sepsis, this issue is either not addressed or not confirmed (J. Pugin, personal communication) [12,16]. However, in the setting of severe community-acquired pneumonia, some authors found that PCT elevation was higher when *S. pneumoniae *was the causative microbe when compared to the so-called atypical agents such as *Legionella *spp [15,23]. In patients with infective endocarditis, PCT elevation was also found to be significantly higher when GN bacteria, rather than GP bacteria, were recovered from blood cultures [14]. These conflicting results might be caused by the fact that previous studies included a broad spectrum of infections, while only proven systemic bacterial infections are considered in the present one. In accordance with our results, some authors have previously shown that in a population with proven sepsis, PCT was significantly higher in patients with bacteremia than in those without [19,24,25]. Differences could therefore be more obvious if only such patients are considered.

Since PCT elevation is deemed to depend directly on inflammatory mediators released by the host in response to the offending pathogen, a different pattern of cytokine response could account for such differences. This hypothesis is supported by the fact that GP and GN bacteria are known to elicit inflammatory responses that rely on different signaling pathways of the innate immunity network. This has recently been illustrated in the blood compartment [26]. Thus, it was shown that the involvement of Toll-like receptors in the whole blood response to various bacterial pathogens was highly variable and depended on the composition of their outer membrane. Composition of the outer membrane is one of the main determinants of the Gram stain result. The magnitude of the cytokine response was thus found to depend on the invading pathogen. More precisely, it has been shown that the Tumor necrosis factor-α (TNF-α) plays a pivotal and very proximal role in the cytokine response to bacteria. However, plasma TNF-α is not necessarily high whatever the causative microorganism may be [27,28]. Given the critical role of this cytokine in the release of PCT from various cell lines in the context of systemic bacterial infection, the magnitude of the PCT elevation could be, at least in part, related to the characteristics of the pathogen. It has been shown *in vitro *that PCT peak value was significantly higher in the supernatants of cultured human cells stimulated with LPS (Lipopolysaccharide) than in those stimulated with muramyl dipeptide, a component of the outer membrane of the Gram positive bacteria [29]. Interestingly, no difference was noted in terms of CRP kinetics. In addition, we have previously shown that bloodstream circulating *Candida *species were less likely to elicit a rise in PCT in the serum of critically ill patients than were bacteria. This could also account for a different pattern of immune response [30].

Our findings should, however, be considered with caution. First, our results could not be generalized to all patients with sepsis since only those with bacteremia were included. Secondly, the likelihood ratio of a positive test (i.e., PCT less than 16.0 ng/mL) is too low to be reliably applied in a clinical setting. Actually, an LR+ equal or superior to 10 is generally required. Thirdly, mortality was significantly higher in patients with GN bacteremia than in those with GP infections. Given the fact that the magnitude of PCT elevation has been shown to be related to the severity of the illness and its prognosis, one should not overlook the fact that patients with GP bacteremia were perhaps less critically ill than their counterparts with GN infections. Although the SOFA score and the admission SAPS II were found to be comparable, there may have been differences in health status [2]. In addition, one cannot exclude the possibility that patients with GP bacteremia were more likely to have been given immunosuppressive drugs. However, no patients in our study were given immunosuppressive drugs other than steroids for septic shock. It is noteworthy that the same proportions of patients in the two groups presented septic shock at the onset of bacteremia and therefore received hydrocortisone. Furthermore, it remains unknown if immunodepressed patients exhibit lower levels of serum PCT in the setting of bacterial sepsis. Finally, it is worth noting that the proportion of soft tissue infections was significantly greater in the GP bacteremia group. As a result, clinical diagnosis and in turn PCT measurement could have been made earlier in those patients, and lower values might have been obtained independently of the Gram stain result. However, similar results were obtained when the patients with soft tissue infections were excluded from the analysis (data not shown). In addition, a low PCT value remains independently associated with GP bacteremia in a model including soft tissue as a source of infection.

## Conclusion

Our findings suggest that baseline PCT elevation could be greater when bacteremia is caused by Gram negative bacteria in comparison with Gram positive bacteria, regardless of the severity of the disease. Since PCT measurement is available sooner than the Gram stain result, its value could be considered when discussing the choice of first line antibiotics in critically ill patients with clinical sepsis. However, clinical findings such as the suspected source of infection as well as the epidemiological context (i.e., community-acquired vs. nosocomial sepsis) are invaluable and should remain the basis for the empiric choice of any antibiotic therapy.

## Competing interests

The author(s) declare that they have no competing interests.

## Authors' contributions

PEC designed the study, analyzed the data and drafted the manuscript. SL collected the data and participated to their interpretation. SA performed the statistical analysis. SL, JPQ, JMD, SP and BB participated to the redaction of the manuscript. NOO managed the activity of the Immunology Laboratory.

## Pre-publication history

The pre-publication history for this paper can be accessed here:


